# Effect of Hyaluronic Acid Filler Injection on the Interdental Papilla in a Mouse Model of Open Gingival Embrasure

**DOI:** 10.3390/ijerph17144956

**Published:** 2020-07-09

**Authors:** Soo-Bin Kim, Jaehun Cho, Seong-Suk Jue, Jae Hyun Park, Ji- Youn Kim

**Affiliations:** 1 Department of Dental Hygiene, College of Health Science, Gachon University, Inchoen 21936, Korea; tnqls11208@naver.com; 2 Department of Oral Anatomy and Developmental Biology, School of Dentistry, Kyung Hee University, Seoul 02453, Korea; 7598119@gmail.com (J.C.); sjue@khu.ac.kr (S.-S.J.); 3 Postgraduate Orthodontic Program, Arizona School of Dentistry & Oral Health, A.T. Still University, Mesa, AZ 85206, USA; jpark@atsu.edu

**Keywords:** black triangle, hyaluronic acid, inflammatory cytokines

## Abstract

The black triangle resulting from interdental papilla (IDP) loss is associated with poor aesthetics and difficulty in pronunciation and food impaction. There is limited knowledge of gingival tissue inflammatory response to hyaluronic acid (HA) filler injection, a minimally invasive IDP reconstruction method. This study aimed to examine the morphological and histological changes in IDP and the inflammatory cytokine localization to the IDP post-HA filler injection using an open gingival embrasure (OGE) mouse model. Mice from the control, sham, and OGE groups were attached with reference, inactive, and activated wires for 5 days, respectively. The degree of IDP loss was determined based on the spring-papilla distance (SPD). Morphological and histological changes in the OGE group injected with phosphate-buffered saline (PBS) or HA fillers were examined on days 2 and 7 post-injection. Immunohistochemical analysis was performed to determine the localization patterns of tumor necrosis factor (TNF)-α, interleukin (IL)-1β, IL-6, myeloperoxidase (MPO), and Ki67. Five days post-wire attachment, the control and OGE groups exhibited a significantly higher SPD than the sham group (*p* < 0.0167). The SPD of the HA filler injection group was significantly lower than that of the PBS injection group on days 2, 4, and 7 post-injection (*p* < 0.05). The IDP of the OGE group was wide and flat. HA filler was stable in the connective tissue underlying the epithelial tissue even on day 7 post-injection. TNF-α, IL-1β, IL-6, MPO, and Ki67 were highly localized to the connective tissue surrounding the filler on day 2, which decreased on day 7 post-injection. Thus, HA filler can safely and successfully reconstruct the IDP in cases of OGE.

## 1. Introduction

The gap between two teeth is filled by the interdental papilla (IDP), a triangular gingival tissue [1]. Various factors contribute to the loss of IDP, such as loss of attached gingiva due to the absorption of alveolar bone, periodontal disease, poor shape reconstruction, and abnormal tooth alignment. The IDP loss-induced gap between the teeth is called a black triangle, which affects tooth aesthetics in the anterior maxillary region and causes various forms of discomfort, such as difficulty in pronunciation and food impaction between the teeth [2]. Therefore, the loss of IDP is an important concern for dentists and patients. Various surgical methods, such as free gingiva and connective tissue graft, and flap design surgery, have been proposed to restore IDP loss. [3]. However, these methods are associated with discomfort to the patient and have limited efficacy [1]. Becker et al were the first to report a minimally invasive IDP reconstruction method using the hyaluronic acid (HA) filler [4]. Several other studies have demonstrated successful reconstruction of IDP using a HA filler [5,6]. Patel et al reported a minimally invasive IDP reconstruction method in humans using a HA filler. The authors demonstrated that the injected filler was stable at eight IDP loss sites for 6 months [7]. Lee et al developed a standardized photographic device for IDP reconstruction and reported that the injected filler was stable for 6 months post-HA filler injection. Of the 43 IDP loss sites, 100% reconstruction was observed in 29 sites and 39–96% reconstruction was observed in 14 sites [8]. Sara et al reported that the HA filler could successfully reconstruct IDP and the reconstruction catered to patient satisfaction [9].

HA, a naturally produced high-molecular-weight substance (105–107 Da), is biodegradable in all organisms [10]. On average, the human body comprises 15 g of HA [11]. HA is involved in various physiological and structural functions [12], such as regulating bleeding and inflammation [13] and promoting bone regeneration and tissue healing [14,15]. HA can exhibit a viscoelastic property upon combination with water in the ratio 1:1000 (v/v). The viscoelastic property of HA contributes to the maintenance of tissue structure and volume [16], structural rigidity, and gap filling [17]. HA is a candidate injectable filler material as it is biocompatible and biodegradable [18]. However, the half-life of HA varies depending on the location in the body (1–3 weeks in the cartilage, 1–2 days in the skin epidermis, and 2–5 min in the blood) [19]. To overcome the short half-life, a cross-linked HA filler has been developed and applied to fill the gap in the human skin, which was found to be stable for 4–12 months [20]. The cross-linked HA filler is prepared from an avian source (rooster comb) or bacteria [21]. The cross-linked HA fillers are broadly divided into animal-based HA or non-animal-based HA [22]. The non-animal stabilized HA (NASHA) filler is produced using bacterial fermentation using a specific strain of *Streptococcus* [22]. Restylane, a NASHA that has been approved by the Food and Drug Administration (FDA) [23], has been widely used in over 60 countries. Although NASHA has several advantages, the injection of NASHA filler into the skin is associated with minor side effects, such as pain, intermittent edema, and erythema [24]. Additionally, some studies have reported temporary and minor side effects associated with NASHA injection, such as discoloration and burning sensation after injection into the IDP loss area [25,26]. Thus, the safety of filler application in the oral cavity, especially gingiva, has not been demonstrated.

Cytokines are inflammatory modulator proteins involved in acute and chronic inflammation [27]. The pro-inflammatory cytokines, which are produced by many cell types, including the macrophages, monocytes, lymphocytes, neutrophils, and fibroblasts, are involved in enhancing the inflammatory response [28]. During the early stages of infection, macrophages, which are involved in inflammatory response against foreign bodies, phagocytose microorganisms [29]. The major pro-inflammatory cytokines include interleukin (IL)-1, IL-6, IL-17, and tumor necrosis factor alpha (TNF-α) [27]. Myeloperoxidase (MPO), a neutrophilic protein, plays a major role in host defense [30]. MPO is expressed in the polymorphonuclear leukocytes and macrophages [31]. The expression of MPO is upregulated in inflammatory lesions [31,32]. Additionally, Ki67, a cell proliferation marker, is expressed in the nucleus during all active phases of the cell cycle (G1, S, G2, and mitosis) but not in the resting cells (G0) [33].

This study aimed to evaluate the safety of intra oral application of HA filler using a mouse model of open gingival embrasure (OGE) through examination of the localization pattern of inflammatory cytokines, such as TNF-α, IL-1β, IL-6, and MPO, in the injected IDP.

## 2. Materials and Methods

### 2.1. Animal

Thirty-five ICR male mice (Orientbio, Seongnam, Korea) were used in this study. The experimental mice were housed under the following conditions: 22 ± 2 °C, 50 ± 5% humidity, and artificial illumination lit between 08:00 to 20:00 h. Food and water were given freely. After wire attachment and injection, mice were fed a normal diet. Experimental protocols were approved by the Gachon University Animal Experimental Ethics Committee (GIACUC-R2019013) and conducted in accordance with the Experimental Animal Center SOP (Standard Operating Procedure).

### 2.2. OGE Model

**Figure 1:**
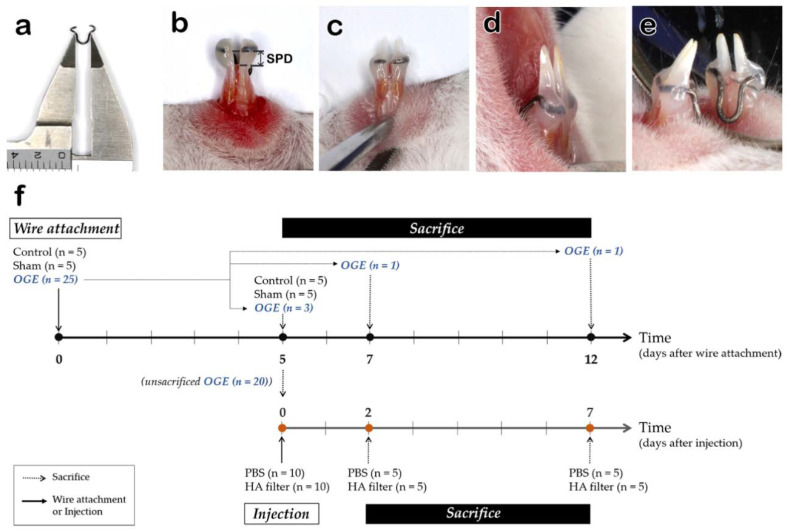
Reproduction of the open gingival embrasure (OGE) model and experimental design. Activated wire (**a**), spring-papilla distance (SPD, arrow) (**b**), attached wire frontal (**c**), lateral (**d**), and lingual (**e**) views. (**f**) Experimental design, showing wire attachment for IDP loss, injection of phosphate-buffered saline (PBS) or hyaluronic acid (HA) filler, and sacrifice schedule.

OGE was modelled through induction of IDP loss in the mouse incisors following the methodology of previous studies [34]. A 9 mm long 0.012″ wire (Australia wire, A.J. Wilcock, Birmingham, England) was used to establish the OGE model. The wire was fabricated to comprise a U-shaped active part and two holding parts surrounding the lateral surface of both incisors (Figure 1). The wire was designed to deliver 50 gf of orthodontic force to both incisors and to move laterally in the distal direction (Figure 1a). The two holding parts were bonded to fit the height of the IDP crest of the mandibular incisors using light-curing composite resin (Transbond™XT Light cure adhesive, 3M Unitek, Monrovia, CA, USA) (Figure 1c–e). A 0.2 mm long reference wire was used for marking the height of the IDP. Inactivation wire had the same shape as the activation wire, but no orthodontic force was generated. The mice were randomly divided into the following three groups: control group (*n* = 5) attached with reference wire, sham group (*n* = 5) attached with inactive wire, and OGE (*n* = 5) attached with activated wire [35]. The animals were anesthetized with an intraperitoneal injection of avertin (0.02 mL/g bodyweight) before attaching the wire. The IDP loss in the OGE group was induced for 5 days and the degree of IDP loss was determined based on the spring-papilla distance (SPD) measured by a Vernier caliper (Figure 1b).

### 2.3. Injection of HA Filler into the IDP

To evaluate the safety of HA filler injection into the IDP loss area, the OGE model mice (*n* = 20) in which IDP loss was induced for 5 days were randomly divided into the following 2 groups: phosphate-buffered saline (PBS) (*n* = 10) and HA filler (Restylane^®,^ Q-Med AB, Uppsala, Sweden) (*n* = 10) injection groups [35]. PBS or HA filler (10 μL) was injected 1–2 mm below the IDP crest after upward the bevel of the syringe with a 30 G needle.

### 2.4. Tissue Preparation

The control (*n* = 5), sham (*n* = 5), and OGE (*n* = 3) groups were sacrificed on day 5 post-wire attachment and subjected to histological analysis. Five mice were sacrificed on days 2 and 7 post-PBS or HA injection, respectively. Additionally, one mouse in the OGE group was sacrificed on days 7 and 12 post-wire attachment as a control of injection groups, respectively (Figure 1f). The mandible was dissected from the mice and immediately fixed in 4% paraformaldehyde (PFA) at 4 °C for 24 h. For decalcification, the sample was incubated with 10% ethylenediaminetetraacetic acid (EDTA, pH 8) for 4 weeks. The specimens were dehydrated and embedded in paraffin. The paraffin-embedded samples were subjected to serial coronal sectioning to obtain 7 µm-thick sections. The sections were dewaxed and rehydrated for hematoxylin and eosin (H&E) and immunohistochemical staining.

### 2.5. Immunohistochemistry

Immunohistochemistry was performed as previously described [36]. The paraffin sections were incubated with the following primary antibodies at 4 °C for 24 h: anti-Ki67 (1:400; Rabbit. no. ab15580; Abcam, Cambridge, UK), anti-TNF-α (1:100; Rabbit. no. ab9739; Abcam, UK), anti-IL-6 (1:500; rabbit. no. NB600-1131; Novus Biologicals, Littleton, CO, USA), anti-IL-1β (1:100; Rabbit. no. ab9722; Abcam, UK), and anti-MPO (1:100; Rabbit. no. ab208670; Abcam, Cambridge, UK). The sections were then incubated with the goat anti-rabbit IgG secondary antibodies (1:500; Rabbit. no. ab6112-HRP; Abcam, Cambridge, UK) at room temperature for 30 min. The sections were counterstained with hematoxylin and mounted on the slides. Each staining per specimen was repeated three times. All sections were observed using a digital microscope (DM2500; Leica, Wetzlar, Germany).

### 2.6. Statistical Analysis

Two investigators measured SPD during the entire experimental period. Intraclass correlation coefficient (ICC) was calculated to estimate the level of agreement between the two measurements. The ICC value was 0.823, which is regarded as indicating good reliability [37]. The degree of IDP loss between the three groups was compared using the Kruskal–Wallis and Mann–Whitney U tests, followed by Bonferroni’s post-hoc analysis (*p* < 0.0167). The differences in SPD value between the HA and PBS injection groups were analyzed using the Mann–Whitney U test (*p* < 0.05). All statistical analyses were performed using SPSS software (SPSS Inc., Chicago, IL, USA).

## 3. Results

### 3.1. Morphological and Histological Changes in IDP after Wire Attachment

**Figure 2:**
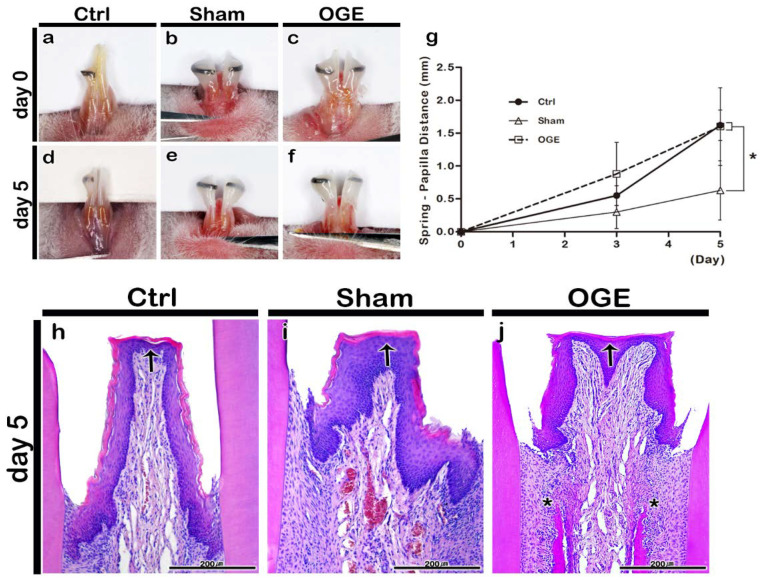
Morphological and histological changes of interdental papilla (IDP) after the wire attachment. Morphology of IDP in control (**a**,**d**), sham (**b**,**e**), and open gingival embrasure (OGE) (**c**,**f**) groups on days 0 (**a**–**c**) and 5 (**d**–**f**) post-wire attachment. Change in spring-papilla distance (SPD) value during the wire attachment period (**g**). The SPD values in OGE and control groups were significantly higher than those in sham group (*p* < 0.0167). Histological analysis of IDP in the control (**h**), sham (**i**), and OGE (**j**) groups after wire attachment. Arrows and asterisks indicate the IDP and high cell density around the alveolar bone, respectively. ^*^ *p*-value was obtained from Kruskal–Wallis test, followed by Bonferroni’s post hoc test (*p* < 0.0167). Scale bars = 200 µm (**h**–**j**).

The bodyweight of mice was measured during the wire attachment period. The bodyweight of mice decreased on day 3 post-wire attachment, but gradually recovered on day 5 post-wire attachment in all three groups. The bodyweight did not decrease by more than 20% of the bodyweight measured at the start of the experiment (data is not shown). There were no deaths or adverse reactions. The SPD value was measured and the images were captured on days 3 and 5 post-wire attachment. In the control group, the position of the reference wire shifted because the incisors erupted during the experimental period (Figure 2a,d). The reference wire was located at the end of the incisor tip on day 5 post-wire attachment. Additionally, the IDP of the control group did not exhibit any morphological changes (Figure 2a,d). The shift in wire position toward the incisor tip was delayed in the sham group when compared with that in the control group. The IDP of the sham group did not exhibit any morphological changes (Figure 2b,e). In the OGE group, the wire position shifted toward the incisor tip after the wire attachment. The IDP of the OGE group was flatter and wider in shape (Figure 2c,f) than the IDP of the sham group. The SPD values increased in the three groups as the incisors erupted. On day 5 post-IDP loss, the SPD values of control and OGE groups were significantly higher than those of the sham group (*p* < 0.0167) (Figure 2g).

The histological analysis of IDP in wire attachment groups was performed using H&E staining on day 5 post-wire attachment. On day 5 post-wire attachment, the keratinized stratified squamous epithelium comprising the basal cell layer, prickle cell layer, granular layer, and keratinized layer, and the underlying connective tissue, were observed in the control, sham, and OGE groups (Figure 2h–j). In the control group, the IDP between the incisors appeared narrow and long (Figure 2h). In the sham group, IDP with a medium width and length was observed (Figure 2i). The IDP in the OGE group was wider and flatter to the left and right when compared with that in the sham and control groups. The separation of junctional epithelium from the tooth surface due to orthodontic force was also observed in the OGE group (Figure 2j). The number of blood vessels and cells in the sham and OGE groups in the connective tissue below the epithelium was higher than that in control group (Figure 2i,j). The cell density around the alveolar bone below the IDP in the OGE group was higher than that in other two groups (Figure 2j).

### 3.2. Morphological and Histological Changes in IDP after PBS or HA Filler Injection

**Figure 3:**
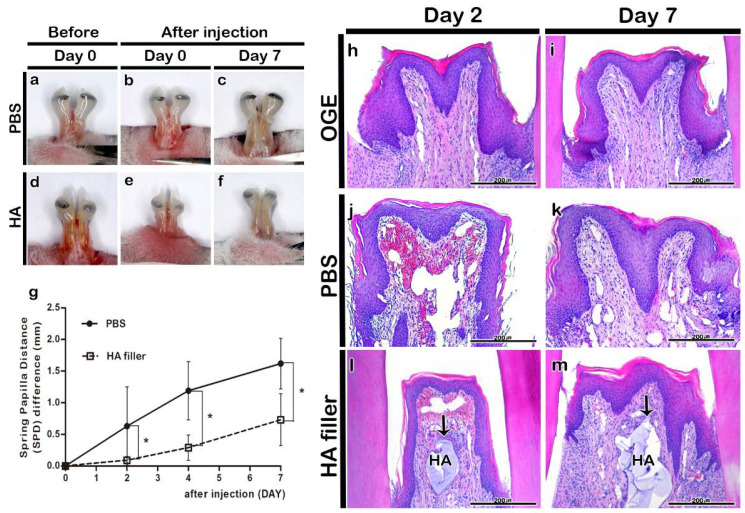
Morphological and histological changes in interdental papilla (IDP) after phosphate buffer saline (PBS) or hyaluronic acid (HA) filler injection. Morphology of IDP in the open gingival embrasure (OGE) group before (**a**,**d**) and immediately after injection (**b**,**e**), and on day 7 (**c**,**f**) post-PBS (**a**–**c**) or -HA filler (**d**–**f**) injection. The spring-papilla distance (SPD) values of the PBS injection group were higher than those of the HA filler injection group on days 2, 4, and 7 post-injection (**g**) (*p* < 0.05). Histological analysis of IDP on days 2 (**h**,**j**,**l**) and 7 (**i**,**k**,**m**) post-injection. Arrows indicate HA filler. HA, hyaluronic acid. ^*^ *p*-value was obtained from Mann–Whitney U test (*p* < 0.05). Scale bars = 200 µm (**h**–**m**).

The morphology of IDP in HA and PBS injection groups was examined to analyze the effect of HA filler on the gingival tissue of OGE group. Injection with PBS or HA filler did not result in hypersensitivity or edema. The injection of PBS or HA filler immediately filled the IDP loss-induced gap (Figure 3a,b,d,e). On day 7 post-injection, the filler was stable in the HA filler injection group even though the incisors erupted (Figure 3c,f). Additionally, the SPD value in the HA filler injection group was significantly lower than that in the PBS injection group on days 2, 4, and 7 post-injection (*p* < 0.05) (Figure 3g).

The histological analysis of IDP in the PBS or HA injection groups was performed using H&E staining on days 2 and 7 post-PBS or -HA filler injection. The OGE group exhibited IDP loss on days 2 and 7 post-injection (Figure 3h,i). The PBS almost disappeared in the PBS injection group. The IDP morphology in the PBS injection group was similar to that in the OGE group on days 2 and 7 post-injection (Figure 3j,k). In the HA filler injection group, the HA filler was observed in the connective tissue under the epithelium of IDP until day 7 post-injection (Figure 3l,m).

### 3.3. Immunolocalization of Ki67 and Inflammatory Cytokines after PBS or HA Filler Injection

**Figure 4:**
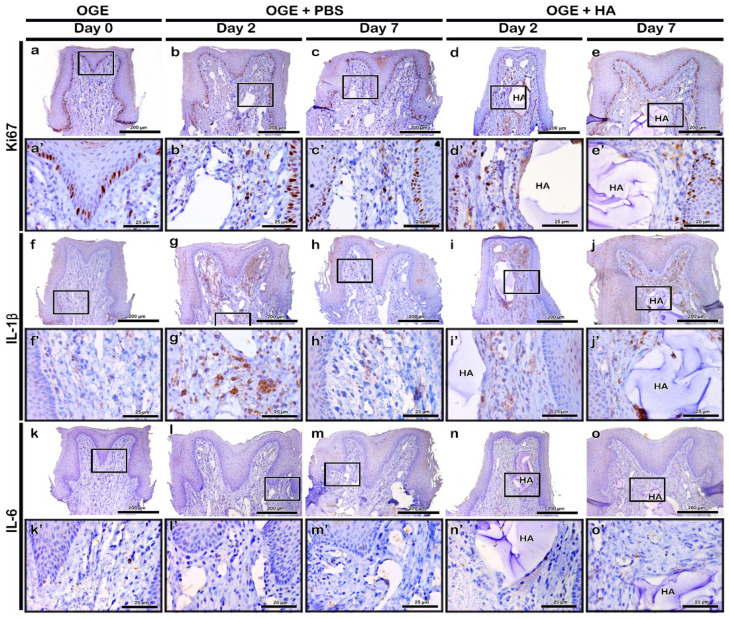
Immunolocalization of Ki67, interleukin (IL)-1β, and IL-6 in interdental papilla (IDP) after phosphate buffer saline (PBS) or hyaluronic acid (HA) filler injection. Localization patterns of Ki67 (**a**–**e**), IL-1β (**f**–**j**), and IL-6 (**k**–**o**) in IDP on days 2 and 7 post-PBS (**b**,**c**,**g**,**h**,**l**,**m**) or -HA filler (**d**,**e**,**i**,**j**,**n**,**o**) injection. Higher magnification view of box in a–o (**a**’–**o**’). HA, hyaluronic acid. Scale bars: 200 µm (**a**–**o**) and 25 µm (**a**’–**o**’).

To evaluate the Ki67 immunolocalization pattern after PBS or HA filler injection in the OGE group, immunohistochemistry was performed on day 5 post-wire attachment and on days 2 and 7 post-PBS or -HA filler injection. Ki67 is a cell proliferation marker. The basal cells of epithelium exhibited obvious Ki67 localization in all three groups (Figure 4a–e). On days 2 and 7 post-PBS injection, Ki67 localization in a few basal cells of the PBS injection group was similar to that of the OGE group (Figure 4a–c). On day 2 post-HA filler injection, Ki67 localization increased in the connective tissue cells around the filler. However, the localization of Ki67 in the HA filler injection group was similar to that in the OGE group on day 7 post-injection (Figure 4d,e).

In the OGE group, IL-1β weakly localized to the connective tissue (Figure 4f). Compared to the OGE group, IL-1β strongly localized to the connective tissue cells on day 2 post-injection in the PBS and HA filler injection groups (Figure 4g,i). On day 7 post-injection, IL-1β exhibited decreased localization to the connective tissue in the PBS and HA filler groups. The localization of IL-1β in PBS and HA filler injection groups was similar to that in the OGE group on day 7 post-injection (Figure 4h,j). IL-6 localized to very few cells of the connective tissue in the PBS and HA filler injection groups, and the OGE group (Figure 4k–o).

**Figure 5:**
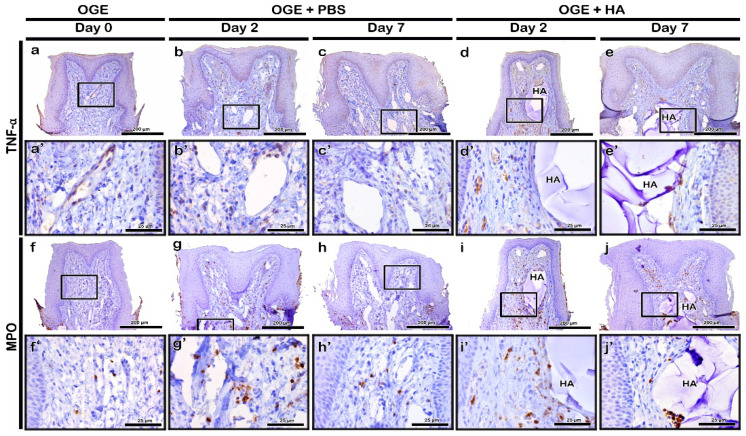
Immunolocalization of tumor necrosis factor (TNF)-α and myeloperoxidase (MPO) in interdental papilla (IDP) after phosphate buffer saline (PBS) or hyaluronic acid (HA) filler injection. The localization patterns of TNF-α (**a**–**e**) and MPO (**f**–**j**) in IDP on days 2 and 7 post-PBS (**b**,**c**,**g**,**h**) or HA filler (**d**,**e**,**i**,**j**) injection. Higher magnification view of box in a–j (**a**’–**j**’). Scale bars: 200 µm (**a**–**j**) and 25 µm (**a**’–**j**’).

TNF-α was highly localized to a few cells of the connective tissue on day 2 post-PBS or -HA filler injection (Figure 5b,d). On day 7 post-injection, the localization of TNF-α to the connective tissue decreased in PBS and HA filler groups. The localization of TNF-α was similar to that in the OGE group on day 7 post-injection (Figure 5a,c,e). MPO rarely localized to the connective tissue in the OGE group (Figure 5f). In the PBS and HA filler injection groups, MPO localized to the cytoplasm of neutrophils in the connective tissue. The localization of MPO was detected in the connective tissue surrounding the injected material on day 2 post-injection (Figure 5g,i). On day 7 post-injection, the localization of MPO decreased in the connective tissue surrounding the injected material (Figure 5h,j).

## 4. Discussion

The black triangle, which is formed due to the loss of IDP, is a major challenge for dentists [1,2]. Various treatments have been proposed to treat this narrow gap and meet the patient’s aesthetic demands [38,39], including the application of HA fillers to the gingiva [4]. Several recent studies have reported the application of HA fillers to the oral cavity [5,6,8]. However, some adverse reactions have been reported after the application of HA filler to the oral cavity [25,26,40]. Therefore, there is a need for precise histological evaluation of IDP to evaluate the safety of intraoral application of HA fillers. In this study, the detailed immunohistological localization patterns of inflammatory cytokines were examined to evaluate the safety of HA filler application at the site of IDP loss.

A previous study successfully developed an experimentally induced OGE model using rats and demonstrated the effect of HA filler injection on IDP reconstruction. However, the study also suggested the need for a histological evaluation to examine the side effects of HA filler injection [34]. Based on the findings of a previous study, OGE was mimicked by inducing IDP loss between both incisors of mice in this study [34]. The morphological changes in IDP of the OGE mouse model were evaluated after wire attachment. In this study, 7-week-old male mice were selected as this age corresponds to the adolescent age, which is associated with active bone metabolism [41]. Additionally, the use of males minimizes the effects of hormones. The wire designed in a previous study was used to model OGE in this study. The active wire, which was designed to continuously apply 50 gf orthodontal force, was used to induce IPD loss for 5 days. The wires were attached to the IDP crest, because the incisors grow continuously in rodents. The degree of IDP loss and the morphological changes in IDP were measured based on SPD, which is the distance from the IDP crest to the wire height. On day 5 post-wire attachment, the SPD values of control and OGE groups were significantly higher than those of the sham group (*p* < 0.0167; Figure 2g). Consistent with the results of previous studies, the sham group exhibited delayed incisor eruption [34,42]. The increase in SPD value in the OGE group indicated successful IDP loss even though the eruption of incisor teeth was delayed (Figure 2). The histological changes in IDP of OGE group were evaluated. Compared to the control and sham groups, the OGE group exhibited the widest IDP. Additionally, the number of blood vessels and cell density around the alveolar bone were high in the OGE group (Figure 2h–j). Upon application of orthodontic force to the teeth, various cellular mechanisms promote periodontal tissue remodeling [43] and affect the blood flow to the periodontal ligament, which induce the secretion of pro-inflammatory cytokines [44]. This suggests that the newly designed wire successfully induced IDP loss.

Various studies have reported the use of fillers as a non-surgical method to fill the gaps in the body [45]. HA fillers are mainly used to increase the volume of the chin, nose, and cheeks, and to provide support to age-related wrinkling [46]. In addition, the effect of HA filler injection into the vocal cords has been examined to restore vocal function in patients with unilateral vocal cord paralysis [47]. Therefore, the fillers must be safe and biocompatible for applications in vivo. The application of fillers should not lead to side effects, such as fever and inflammation, and should not exhibit antigenicity or toxicity. Additionally, the fillers should be stable in the body after injection [48,49]. However, several studies have reported a temporary redness and swelling reaction after injection of the HA filler into the face. The reaction appears to be due to the immune response caused by foreign body invasion in vivo, but disappears after a few days [45]. Inflammation is a defense mechanism to eliminate harmful stimuli and initiate a healing process. When inflammation occurs, many immune cells release various inflammatory mediators. It causes blood vessels to become dilated and increase blood flow, resulting in heat and redness [50]. Therefore, this study examined the effect of Restylane, an FDA-approved HA filler, injected into the site of IDP loss, on the morphology and histology of IDP. The SPD value in the HA filler injection group was significantly lower than that in the PBS injection group (*p* < 0.05) (Figure 3g). In the HA filler injection group, HA filler was observed in the connective tissue under the epithelial tissue even on day 7 post-injection (Figure 3m). Immunohistochemical analysis of Ki67, a cell proliferation marker, revealed that the cells in the basal cell layer of stratified squamous epithelium exhibited active proliferation. The Ki67-positive area in connective tissue around the filler increased on day 2, but decreased on day 7 post-injection (Figure 4a–e). Injection of HA filler is reported to induce collagen synthesis, which increases collagen deposition around the filler [46,51,52]. Injection-induced mechanical stress is reported to activate the fibroblasts and enhance the production of extracellular components [51,53]. The inflammatory cytokines are secreted by immune cells including lymphocytes, macrophages, helper T cells, and other cell types that promote inflammation [28]. The degree of immune cell infiltration, including of neutrophil, is an important indicator of the initial degree of inflammation [54]. In the OGE group, IL-1β, IL-6, and TNF-α weakly localized to the connective tissue of IDP (Figure 4f,k and Figure 5a). Orthodontic treatment is associated with an acute inflammatory response [55], which is consistent with the results of previous studies that reported increased levels of IL-1β and TNF-α in the periodontal tissues after the application of orthodontic force [56,57]. In the HA filler injection group, TNF-α and IL-1β (known as pro-inflammatory cytokines) strongly localized around the filler on day 2 post-injection. The localization patterns of TNF-α, IL-1β, and IL-6 in the HA filler injection group were similar to those in OGE group on day 7 post-injection (Figure 4f–o and Figure 5a–e). Previous studies have reported that the phagocytic cell induction observed after injecting HA fillers into the skin is an immune response to exogenous foreign bodies in vivo [58]. The acute inflammatory reactions secrete pro-inflammatory cytokines such as TNF-α, IL-1β, and IL-6 as a primary defense against tissue damage and secrete inflammatory transmission materials that cause inflammation [59]. In both PBS and HA filler injection group, immunolocalization of inflammatory cytokines initially increased (Table 1). This suggests that the initial immune response might occur as a reaction to the needle or orthodontic force used to induce IDP loss. Mild or moderate inflammation might be considered as a clinical benefit for tissue regeneration. In addition, previous studies have reported that the HA filler does not promote granuloma formation and does not affect the fibroblast metabolic activity [58]. This indicates that the HA filler can be safely used for clinical applications, particularly for gingival tissues, without adverse effects.

A large number of red blood cells was observed in the connective tissue around the filler after HA filler injection (Figure 3j,l). The blood vessels undergo damage in mice because the IDP area in mice is narrower than that in humans and because injection requires expert handling. Lee et al reported that the use of an automatic injector enables the injection of a small amount of HA filler into the site of IDP loss, decreased shaking of the needle, and accurate injection [8]. Therefore, the use of an auto-injector in clinical settings will minimize variations caused due to differences in the skill level of the operator.

This is the first study to assess the immunolocalization pattern of inflammatory cytokines in the IDP loss site injected with HA filler. This study has some limitations. The observation period of this study was short (7 days). The mechanism underlying HA filler biodegradation in the oral cavity is unknown. Therefore, further studies are needed to evaluate the long-term stability of HA filler in the oral cavity, and to evaluate the mechanism underlying the biodegradation and persistence of HA filler in the oral tissue. However, this study successfully induced IDP loss in mice and demonstrated morphological and histological changes in IDP after HA filler injection. Additionally, the localization pattern of inflammatory cytokines after HA filler injection was elucidated. The temporal localization of inflammatory cytokines demonstrated that HA injections to fill the IDP loss-induced gap elicited a temporary immune response that disappeared naturally within days. Therefore, the findings of this study suggest that HA filler can be used as a safe candidate material for reconstructing IDP in cases of OGE.

## 5. Conclusions

This study demonstrated an in vivo model of IDP loss and the reconstruction of the IDP loss-induced gap through HA filler injection. The inflammatory cytokines, such as IL-1β, IL-6, TNF-α, and MPO, localized to the IDP after HA filler injection. The initial inflammation response disappeared within days. This suggests that the initial inflammation can be induced by mechanical stress such as orthodontic force or needle and might be reduced by injected HA filler. Therefore, the findings of this study indicate that intra-oral HA filler application is a minimally invasive and safe IDP reconstruction method. HA filler is a safe material that can be used for the reconstruction of IDP in cases of OGE.

## Figures and Tables

**Table 1:** The immunolocalization pattern of inflammatory cytokines in the interdental papilla loss site injected with phosphate-buffered saline (PBS) or hyaluronic acid (HA) filler.

Antibody	Marked Cell	Localization Pattern			
		PBS Injection		HA Filler Injection	
		Day 2	Day 7	Day 2	Day 7
**Ki67**	Cell proliferation/nuclear	Basal cells of epithelium, Connective tissue around the injected material	Basal cells of epithelium	Basal cells of epithelium, Connective tissue around the injected material	Basal cells of epithelium
**IL-1β**	Macrophages/fibroblasts	Connective tissue cells	-	Connective tissue cells	-
**IL-6**	Macrophages/fibroblasts	Very few cells in connective tissue			
**TNF-α**	Activated macrophages	Connective tissue cells	-	Connective tissue cells	-
**MPO**	Neutrophils	Connective tissue around the injected material	-	Connective tissue around the injected material	-

IL, interleukin; TNF, tumor necrosis factor; MPO, myeloperoxidase.
